# Rapamycin increases grip strength and attenuates age-related decline in maximal running distance in old low capacity runner rats

**DOI:** 10.18632/aging.100929

**Published:** 2016-03-19

**Authors:** Qian-Li Xue, Huanle Yang, Hui-Fen Li, Peter M. Abadir, Tyesha N. Burks, Lauren G. Koch, Steven L. Britton, Joshua Carlson, Laura Chen, Jeremy D. Walston, Sean X. Leng

**Affiliations:** ^1^ Department of Medicine Division of Geriatric Medicine and Gerontology, Johns Hopkins University, Baltimore, MD 21205, USA; ^2^ Center on Aging and Health, Johns Hopkins Medical Institutions, Baltimore, MD 21205, USA; ^3^ Department of Anesthesiology, University of Michigan, Ann Arbor, MI 48109, USA; ^4^ Department of Molecular and Integrative Physiology, University of Michigan, Ann Arbor, MI 48109, USA

**Keywords:** rapamycin, low capacity runner rats, aging, healthspan, physical function

## Abstract

Rapamycin is known to extend lifespan. We conducted a randomized placebo-controlled study of enteric rapamycin-treatment to evaluate its effect on physical function in old low capacity runner (LCR) rats, a rat model selected from diverse genetic background for low intrinsic aerobic exercise capacity without genomic manipulation and characterized by increased complex disease risks and aging phenotypes. The study was performed in 12 male and 16 female LCR rats aged 16-22 months at baseline. The treatment group was fed with rapamycin-containing diet pellets at approximately 2.24mg/kg body weight per day and the placebo group with the same diet without rapamycin for six months. Observation was extended for additional 2 months. Physical function measurements include grip strength measured as maximum tensile force using a rat grip strength meter and maximum running distance (MRD) using rat physical treadmill test. The results showed that rapamycin improved grip strength by 13% (p=.036) and 60% (p<.001) from its baseline in female and male rats, respectively. Rapamycin attenuated MRD decline by 66% (p<.001) and 46% (p=.319) in females and males, respectively. These findings provide initial evidence for beneficial effect of rapamycin on physical functioning in an aging rat model of high disease risks with significant implication in humans.

## INTRODUCTION

Co-existence of complex multiple chronic conditions is rather a norm than exception in older adults. As such, interventional strategies that improve overall health and function, or healthspan, above and beyond disease-specific benefit and longevity have become critically important for this growing and yet vulnerable population as well as for the society [1, 2]. Animal models with diverse genetic background suitable for the study of complex disease risks and aging may facilitate developing such strategies and are of immediate interest to the field of gerontology. While such ideal model systems with no genomic manipulation are scarce, a rat model of intrinsic aerobic exercise capacity can serve at least to some extent, as one. This rat model was developed from the genetically heterogeneous N:NIH out-crossed stock [3] through 2-way selective breeding using a rotational breeding scheme on the trait of running capacity measured by maximal distance run to exhaustion on a speed-ramped treadmill [4]. Low capacity runner (LCR) rats derived from generations 14, 15, and 17 demonstrated 7-fold lower average aerobic running capacity, up to 45% shorter lifespan than high capacity runners (HCRs) [5]. LCRs also display increased complex metabolic and cardiovascular disease risks, increased sensitivity to fatigue, and a number of aging phenotypes including decreased level of physical activity, reduced mitochondrial regeneration and antioxidant status, as well as a pro-inflammatory state [5-10].

Rapamycin, a mammalian target of rapamycin (mTOR) inhibitor has been demonstrated to extend lifespan in a range of model organisms. It is believed that rapamycin extends longevity likely through suppressing cancers (reviewed by Ehninger D, et al. [11]). While still being debated, rapamycin may slow mammalian aging or improve healthspan as some studies have shown positive impact of rapamycin and, more recently, rapalogs (such as RAD001) on age-related cognitive impairment, immunesenescence, and overall health in mice or humans [12-15]. The objective of this study was to evaluate the effect of rapamycin on age-related decline of physical function in old LCR rats. We hypothesized that rapamycin would ameliorate or slow such functional decline over time. To test this hypothesis, we conducted a randomized, placebo-controlled study of enteric rapamycin-treatment in male and female LCR rats aged 16-22 months. The duration of rapamycin-treatment was 6 months with observation extended for an additional two months.

## RESULTS

### Baseline characteristics of the study animals and blood rapamycin levels in rapamycin-treated group

Table 1 lists baseline characteristics of the LCR rats at study entry. Female rats on average weighed 335.3g [standard deviation (SD)=47.4], had mean grip strength of 1,338g (SD=160) and MRD of 128.5m (SD=28.6). In contrast, male rats were considerably heavier (mean=600.4g; SD=82.4), had greater grip strength (mean=1,418, SD=227) and shorter MRD (mean=41.8m, SD=31.4) than females. In order to account for the age difference between males (age 20-22 months) and females (age 16-18 months) at study entry, we also derived summary statistics for female placebo rats at 20-22 months of age. Compared to all male rats at 20-22 months of age, female rats of the same age on average weighed 365.5g [standard deviation (SD)=31.3], had mean grip strength of 1,513g (SD=121) and MRD of 72.4m (SD=41.6). The sex differences at 20-22 months of age in grip strength and MRD were statistically significant after accounting for sex differences in body weight (p<0.001 for adjusted grip strength; p=0.002 for adjusted MRD; results not shown). The distributions of the study measures among rats of the same sex were not statistically different between rapamycin vs. placebo groups at study entry (Table 1).

**Table 1:** Mean (standard deviation) of physical function and body weight of male and female LCR rats by treatment assignment at study entry

	Male (age 20-22 months)			Female (age 16-18 months)		
Outcome Measures	Overall (n=12)	Rapa.-treated (n=6)	Placebo (n=6)	Overall (n=16)	Rapa.-treated (n=8)	Placebo (n=8)
Grip strength (g)	1418 (227)	1442 (278)	1394 (185)	1338 (160)	1322 (139)	1355 (187)
Adjusted Grip Strength (g/100g weight)	239 (39)	244 (37)	233 (43)	406 (65)	398 (59)	413 (74)
MRD (m)	41.8 (31.4)	50.0 (35.2)	33.6 (27.7)	128.5 (28.6)	138 (25)	119 (30)
Adjusted MRD Ln(m/100g weight)	1.64 (0.98)	1.90 (0.87)	1.39 (1.09)	3.63 (0.30)	3.71 (0.28)	3.56 (0.31)
Body Weight (g)	600.4 (82.4)	590.7 (66.2)	610.2 (101.5)	335.3 (47.4)	338.9 (58.8)	331.8 (36.4)

Rapa: rapamycin; MRD: Maximum running distance; Ln: natural logarithm

Overall, there was no significant difference in food intake between rapamycin-treated and placebo groups in male or female rats (data not shown). Average blood rapamycin levels in rapamycin-treated group were 1.96ng/ml at week 4 and 6.49ng/ml at week 16 for male rats and 1.65ng/ml at week 10 and 2.82ng/ml at week 18 for females. These levels, while lower than those found in a mouse longevity study (60-70ng/ml) [16], are comparable to the reported 3-5ng/ml in C57BL/6J mice at both young (10 months) [15] and old (19 months) [17] age after 6 months of enteric rapamycin treatment; and the level increased over time indicating rapamycin intake and absorption.

### Primary Outcomes

#### Grip Strength Trajectory

**Figure 1:**
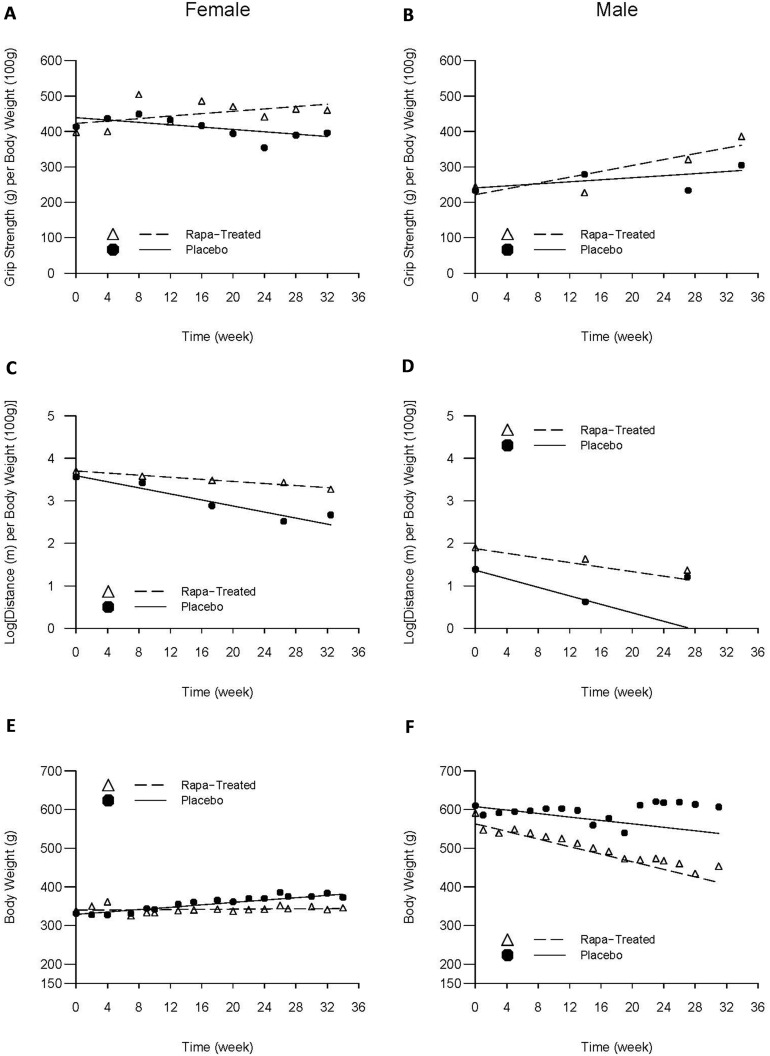
Average trajectories of grip strength, maximum running distance, and body weight over time by intervention status and sex The dashed and solid lines respectively represent rapamycin-treated and placebo group-specific mean trajectories estimated from the linear random effects model that took into account the premature deaths; the triangles and solid cycles respectively represent rapamycin-treated and placebo group-specific cross-sectional means based solely on the survivors at the time of each evaluation.

For female LCRs, body-weight adjusted grip strength increased at an average rate of 6.7g/month in rapamycin-treated group (p=0.036), compared to a trend of 6.7g/month decline in the placebo control group (p=0.062); and the difference was highly significant (p=0.005). Adjusted grip strength of the male LCRs increased at an average rate of 18.0g/month in rapamycin-treated group (p<0.001), compared to a trend of 6.3g/month increase in the placebo group (p=0.132); and the difference was also statistically significant (p=0.043) (Figure 1A, B).

#### MRD Trajectory

Overall, females' treadmill performance worsened over time with adjusted MRD declining linearly with age. However, the rate of decline among rapamycin-treated females was two-thirds less compared to the placebo group (0.052 vs. 0.155 Ln(m)/month, respectively, p<.001). The same trend was observed in the male rats, although it did not reach statistical significance: rapamycin-treated males experienced an average decline of 0.117 Ln(m)/month compared to 0.219 Ln(m)/month, representing a 46% reduction relative to their placebo counterparts (p=0.319) (Figure 1C, D).

### Secondary outcomes

#### Mortality

During the 8-month follow-up, 3 (50%) males (on day 9, 192, 203) died and all females survived in rapamycin-treated group and 3 (50%) males (on day 95, 100, 127) and 3 (38%) females (on day 143, 175, 179) died in the placebo group. Compared to their placebo counterparts, the Cox model showed a 16% reduction in mortality in the rapamycin-treated males, after accounting for time of death and adjusting for age (p=0.83). It is also worthwhile noting that all females in rapamycin-treatment group survived while there was a 38% mortality among females in the placebo group.

#### Weight Trajectory

Among females, while there was little change in body weight over time in rapamycin-treated group (0.54g/month; p=0.722), body weight increased significantly at a rate of 6.7g/month in the placebo group (p<0.001). In contrast, male rats experienced weight loss of 9.7g/month in the placebo group (p=0.144) and 21.3g/month in rapamycin-treated group (p=0.002); the difference in the rate of weight loss between the two groups of males, however, was not statistically significant (p=0.231) (Figure 1E, F).

### Side effects of rapamycin-treatment

Approximately four months into the first experiment, two of the remaining 5 male rats in the treatment group developed diabetes-like symptoms (increased food intake, weight loss and frequent urination). Blood glucose test was done on all male survivors (5 on treatment and 3 on placebo) at age of approximately 27-29 months after 17-hr fasting. The average glucose level was 143.4mg/dl (SD=67.1) in the treatment group and 100 (SD=21.4) in the placebo group (p=0.237). Two rapamycin-treated male rats with diabetic symptoms described above had glucose level >150mg/dl on repeated tests. Their food consumption increased respectively by 66% and 92% with accompanying weight loss of 26% and 9% after 6 months of rapamycin treatment; and their blood rapamycin concentration levels were respectively 14.9 and 3.84ng/ml after four months of rapamycin treatment. None in the placebo group had glucose level >150mg/dl.

In the second experiment, none of the rapamycin-treated female rats or those in the placebo group showed any diabetic symptoms or had fasting glucose exceeding 100mg/dl over the course of the study.

## DISCUSSION

This randomized placebo-controlled study observed, for the first time, that enteric rapamycin-treatment led to improvement of grip strength and better maintenance of MRD over time in old LCR rats, a rat model characterized by increased complex disease risks and aging phenotypes. Although this study was not powered to detect significant difference in mortality (as a secondary outcome) between treatment and placebo groups, the observed improvement in lifespan among the treated, while not statistically significant, is consistent with findings in other vertebrate and invertebrate model organisms [18].

Significant sex differences were noted in body weight, grip strength and MRD at the study entry, and these baseline differences might be due, in part, to differences in age as the male rats were 4-6 months older than the females. However, the differences remained significant even after accounting for the age difference. Sex differences were also observed in rapamycin treatment-related weight changes, diabetogenic effects, and survival, which are consistent with observations in other animal studies [15, 16, 19, 20] and in humans (diabetes as a side effect of rapamycin-based cancer therapy) [21]. Moreover, responses to rapamycin-treatment in grip strength and MRD were more robust in females. It has been hypothesized that these sex differences could result from sex-specific differences in rapamycin absorption into the blood stream [20], different sensitivity to rapamycin [22], and sex-specific changes in mTOR signaling with age in mice [23]. Whether these are also true in rats and can account for the observed sex differences remains unclear. Further investigation into the regulation of mTOR signaling and its relationship with functional outcomes in this LCR rat model is currently underway.

We selected aged LCR rats as a non-disease-specific model for the study. This is because in apparently healthy animals, one recent study found no effect of rapamycin treatment on age-related changes in grip strength and gait performance [15]. In that study, rapamycin-treatment resulted in no improvement in movement in either male or female mice [15]. Moreover, Neff et al. reported that rapamycin-treatment did not ameliorate age-associated grip strength decline or improve oxygen consumption [24]. These discrepancies may point to the need of targeting rapamycin-treatment to an animal model that already manifests metabolic dysregulation and aging phenotypes. Evidence supporting this hypothesis includes rapamycin treatment-related decrease in obesity and prevention of weight gain in rodents and humans [25-32] as well as downregulation of fasting levels of phosphorylated S6 (p-S6; a marker of mTORC1 activity) in mice on high-fat diet [33, 34]. Given that high fasting levels of p-S6 is indicative of increased geroconversion from quiescence to senescence [35, 36] and the suppression of geroconversion by rapamycin prevents age-related diseases in mammals [34], the observed beneficial effects on age-related declines in muscle strength and aerobic capacity among LCR rats exhibiting metabolic dysregulation may in fact result from rapamycin-induced suppression of “systemic hyper-functions (e.g. pro-inflammtory conditions) associated with aging [37]. Alternatively, the discrepancies may be related to different species, inbred vs. outcrossed animals, or other study variables. Nevertheless, findings from our study provide initial evidence for beneficial effect of 6-month enteric rapamycin-treatment on grip strength and MRD of the LCR rats initiated relatively late in life. Such findings have important clinical implication as they provide a basis for potential development of rapamycin-based interventional strategy to delay or reverse late-life functional decline and to improve quality of life for older adults.

Limitations for this pilot study include small sample size, inability to draw conclusions on specific mechanisms underlying the observed effects, and lack of body composition measures such as lean vs. fat mass. Because of the influence of body weight on the measurement of MRD and grip strength as evidenced by the positive correlation between body weight and grip strength (Pearson's correlation (ρ)=0.31; p-value=0.103) and negative correlation between body weight and MRD (ρ=−0.86; p-value<0.001) at baseline, we normalized MRD and grip strength in the analyses in order to distinguish the effect of rapamycin treatment on function from the influence of weight on the measurement. This study has several strengths. First, the selection of grip strength and MRD as primary outcomes was motivated by not only their functional significance for maintaining independence in late life but also their mechanistic links to age-related decline in mTOR-mediated muscle protein turnover [38], therefore providing a hypothesis-driven mechanistic framework by which to distinguish causal relationships from non-causal associations. Secondly, the study was conducted in an investigator-blinded fashion with the same food pellets and nutrients for study animals except for rapamycin. This was to model after double-blind, placebo-controlled trial in humans and further ascertain causal relationships between rapamycin-treatment and observed phenotypic alterations. Lastly, the longitudinal design with repeated measurements spanning 8 months of late life in rats provided a rare opportunity to assess the health effects of rapamycin in an animal model that already has metabolic dysregulation and aging phenotypes. Taken together, despite the stated limitations, results from this study support our original hypothesis and provide initial evidence for the beneficial effect of enteric rapamycin-treatment on physical functioning in addition to lifespan in a non-disease specific aging animal model. More research is indicated to further expand these findings and elucidate the mechanism(s) by which rapamycin improves physical function and healthspan.

## MATERIALS AND METHODS

### Animals and treatment groups

Twelve male LCR rats from generation #26 and 16 female LCR rats from generation #29 were used in the study via two experiments conducted in sequence beginning with the males. The rats are developed and maintained as a research resource at the University of Michigan (S.L.B. and L.G.K.). Females and males were 16-18 months and 20-22 months old, respectively, at study entry.

Rapamycin-containing food pellets at the concentration of 14 mg per kg of food, along with placebo food pellets which were constructed in the same way with the same physical appearance and nutrients without rapamycin, were purchased from Southwest Research Institute (San Antonio, Texas). The treatment group consumed rapamycin-containing food pellets with rapamycin intake approximately 2.24 mg/kg body weight per day as previously reported [16]. Male and female rats were randomized into two groups consuming either rapamycin-containing food pellets or placebo. To monitor rapamycin intake and absorption, blood rapamycin levels were measured in the treatment group using mass spectrometry at the Biological Psychiatry Analytical Lab, University of Texas at San Antonio as previously described [16]. Table 2 details the study variables and data collection timeline. Tests and data collection were conducted by the same evaluator (H.Y.) who was blinded to treatment assignment. All experimental procedures were approved by Johns Hopkins University Institutional Animal Care and Use Committee.

**Table 2:** Randomized, placebo-controlled study of enteric rapamycin-treatment in old LCR Rats: Study variables and data collection timeline

	Timeline (Week)																
	0	2	4	6	8	10	12	14	16	18	20	22	24	26	28	30	32
**Rapamycin feeding**	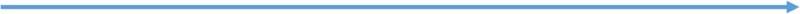																
**Study Measure**																	
Grip Strength	♂/♀		♀		♀		♂/♀		♀		♀		♂/♀		♀		♂/♀
MRD	♂/♀				♀		♂		♀				♂/♀		♂		♀
Body Weight	♂/♀	♂/♀	♂/♀	♂/♀	♂/♀	♂/♀	♂/♀	♂/♀	♂/♀	♂/♀	♂/♀	♂/♀	♂/♀	♂/♀	♂/♀	♂/♀	♂/♀
Blood Rapamycin Levels			♂*			♀#			♂*	♀#							
Food Intake	♂/♀	♂			♀	♂			♂/♀				♀				
Fasting Glucose	♀		♀	♀	♀	♀	♀	♀	♀	♀	♀		♂/♀				♀

MRD: Maximum running distance

* Measured in a random sample of 3 rapamycin-treated male rats at 4 weeks and 4 rapamycin-treated male rats at 16 weeks.

# Measured in all 8 rapamycin-treated female rats at 10 weeks and 7 rapamycin-treated female rats at 18 weeks.

### Measurements of physical function

#### Grip Strength

Fore-limb grip strength was measured as maximum tensile force every three months for males and monthly for females using a rat Grip Strength Meter (Columbus Instruments, Columbus, OH) with a sensor range of 0–5,000grams (g), and accuracy of 0.15% [39]. The maximum of three pulls of the Grip Strength Meter conducted between 9am and 11am was recorded; the test was performed daily over three consecutive days and the resulting three daily maximum values were averaged.

#### Maximum Running Distance (MRD)

Measurement protocol was adapted from the one routinely employed at University of Michigan as previously described [4]. Briefly, a rat physical test treadmill with electric shock apparatus at the end of the running track was utilized (Columbus Instruments, Columbus, OH). Rats were individually trained for 3 days immediately preceding data collection by gradually increasing treadmill start speed and incline. Final test was conducted on the treadmill with an incline of 15 degrees and start speed at 5m/min and an acceleration interval of 0.5m/min every two minutes. An electric shock was given at a constant intensity of level 7 (max. 10) with a 3Hz repetition rate to the animal upon falling off the running track. The test was stopped when cumulative shocks reached 18 for both males and females. For each evaluation, the test was repeated on 3 consecutive days between 9am and 3pm. For each running trial, total running time and distance on track were recorded, and average of MRD in meters (m) over the three trials were used in the analysis.

### Weight, mortality and other measurements

Weight was measured every two weeks using a PB3002 DeltaRange balance (Mettler Toledo, Switzerland). Mortality and food intake were monitored over time according to standard animal and laboratory methods. Fasting glucose was measured from whole blood collected from saphenous vein using test strips and the Bayer Contour Blood Glucose Meter (Bayer Healthcare LLC, Mishawaka, IN 46544).

### Statistical analysis

To take advantage of the longitudinal data and reduce bias in treatment effect estimation due to premature death, linear random effects models (REM) [40] were used to assess the effects of rapamycin on changes in grip strength, MRD, and body weight over time. The REM models the individual trajectories of each study variable by including subject-specific baseline value and rate of change per month of the study variable as random effects. The REM adjusts for within-subject correlation in the repeated measurements over time through an unstructured correlation matrix for the random effects. By averaging the subject-specific trajectories within experimental groups, the REM was able to provide group-specific mean trajectory that is robust to data missing at random (i.e., the propensity for a data point to be missing can be reliably predicted by the observed data) [41]. For example, missing data due to death are missing at random if those who died prematurely demonstrated faster declines in physical function compared to the survivors, which is a reasonable assumption to make in this study. To adjust for the confounding effect of body weight on grip strength and running capacity, the ratios of strength (g) and MRD (m) to body weight (g) were used as adjusted outcome measures and are reported in units of grams (strength) or meters (MRD) per 100g of body weight. Adjusted MRD value was natural logarithm-transformed [Ln(m)] to approximate normality in order to maximize inferential validity.

## Abbreviations

LCR: low capacity runner
MRD: maximum running distance
mTOR: mammalian target of rapamycin
